# Impact of Oxidative Stress and Antioxidants on Semen Quality in Dogs

**DOI:** 10.3390/ani15213169

**Published:** 2025-10-31

**Authors:** Sławomir Zduńczyk, Anna Domosławska, Monika Jamioł, Marta Kankofer

**Affiliations:** 1 Department of Animal Reproduction with Clinic, Faculty of Veterinary Medicine, University of Warmia and Mazury in Olsztyn, 10-719 Olsztyn, Poland; zdun@uwm.edu.pl (S.Z.); anna.domoslawska@uwm.edu.pl (A.D.); 2 Department of Biochemistry, Faculty of Veterinary Medicine, University of Life Sciences in Lublin, 20-033 Lublin, Poland; monika.jamiol@up.lublin.pl

**Keywords:** dog, semen, oxidative stress, antioxidant supplementation

## Abstract

**Simple Summary:**

Canine sperm are sensitive to oxidative damage. The article reviews antioxidant defense in canine semen and the potential role of antioxidant supplementation of male dogs and semen extenders in improving sperm quality and fertility.

**Abstract:**

This review focuses on the biochemical mechanisms of oxidative damage to sperm, the role of antioxidants, as well as the clinical consequences of supplementation for the improvement of sperm fertility. There is growing interest in dog breeding and in methods of maintaining semen quality for natural mating or artificial insemination (AI). Canine sperm are sensitive to oxidative damage. Semen contains endogenous, enzymatic, renewable, and non-enzymatic, and non-renewable antioxidants. However, the excess of reactive oxygen species (ROS) or depletion in antioxidative defense may lead to oxidative stress, causing damage to sperm cells and a decrease in fertility. The possible way to maintain sperm cell fertility potential is supplementation of diet and/or semen extenders with antioxidants. It seems that oral antioxidant supplementation improves the oxidative status and quality of semen and may have a positive effect on the fertility of male dogs with reproductive problems. Many studies point to the potential role of antioxidant supplementation in extenders in protecting canine sperm from oxidative damage during processing. However, only a few studies have assessed the fertilization capacity of supplemented sperm after AI. Intensive studies, including the examination of pro- and antioxidative properties of dog semen as well as the role of antioxidant supplementation to dogs and semen extenders, should be performed, as it is a serious market and breeders need. The results should be related not only to semen analysis but pregnancy rate as the best marker of fertility. Nevertheless, the use of antioxidants in the supportive treatment of fertility disorders in male dogs to improve semen quality and their addition to dog semen extenders to preserve sperm fertility appears to be reasonable.

## 1. Introduction

In recent years, current needs in breeding of different breeds of dogs have forced the control of semen quality and its improvement. Due to economic reasons, breeders expect pregnancy at every mating, which is only possible when both bitch and dog are healthy and have appropriate reproductive potential. While small quantities of ROS are necessary for capacitation of sperm cells, their excess or depletion in antioxidative defense may lead to oxidative stress [1]. Oxidative stress causes serious biochemical consequences, such as the decrease in adenosine triphosphate (ATP) concentrations, the increased catabolism of adenine nucleotides, diminished NAD supply, the decrease of reduced (GSH) and oxidized (GSSG) glutathione ratio (GSH/GSSG ratio) as well as total glutathione, and the increase of Ca^2+^ content by the inactivation of calcium pump and activation of proteases. All macromolecules undergo peroxidative damage, leading to alterations in cell membrane permeability, the decrease or inhibition of enzyme activity, as well as changes in DNA structure responsible for mutations [2].

Antioxidants in semen are divided between sperm cells and seminal plasma. The classic triad of enzymatic antioxidants, comprising superoxide dismutase (SOD), glutathione peroxidase (GSH-Px), and catalase (CAT), is mainly located intracellularly in sperm cells and supported by not only non-enzymatic antioxidants but also by glutathione reductase or thioredoxins [3]. Non-enzymatic antioxidants are mainly present in seminal plasma. Among classic non-enzymatic and non-reusable antioxidants are water-soluble glutathione, uric acid, vitamin C, cysteine, bilirubin, carnosine, as well as lipid-soluble vitamin A, E, and derivatives of estrone and oestradiol [1].

As the majority of antioxidants are of protein origin, new proteomic techniques can be implemented in the analysis of not only seminal plasma but also sperm cells. This analysis provides knowledge about new proteins that may support the antioxidative system, which were not known before. Moreover, new protein molecules serving as biomarkers can be discovered. Transcriptomic analysis of these proteins may uncover their genes and a possible source of disturbances on the genetic level.

A scientific hypothesis was stated that not only pathogens or pathological processes but also environmental factors may influence semen quality and fertility. Among such dangers may be cigarette smoke [4], heat stress [5], or a wrong diet [6], which causes oxidative stress and may influence antioxidative defense. Even if there are limits for smoking in public areas, dogs can be passive smokers. Handling and preservation of semen may also cause sperm cell damage due to, among others, oxidative stress.

Animals can be supplemented with antioxidants in the diet, which improves the general antioxidative system. Moreover, extenders used for semen preservation can be enriched in antioxidants and help maintain semen quality. Endogenous antioxidants are used, but also exogenous molecules known for their antioxidative properties are tested.

This review will cover the following:

- The review of current literature about enzymatic and non-enzymatic antioxidants in sperm cells and seminal plasma of dogs, including proteomic aspects,
- Disorders of semen quality leading to infertility related to disturbances in pro- and antioxidative balance in dogs,
- The summary of studies on antioxidant supplementation of dogs and extenders for canine semen,
- Limitations of current data and perspectives for the future in dogs.

## 2. Materials and Methods

A systematic literature search was conducted in August and September 2025 using the following electronic databases: PubMed and Google Scholar. The search was limited to peer-reviewed scientific articles published in English. The literature search was conducted using the following keywords: male dogs, oxidative stress, antioxidant, semen, and fertility. The collected articles were thoroughly evaluated for their potential relevance. Only studies published as full-length, peer-reviewed scientific articles were included.

## 3. Antioxidant System in Semen of Dogs

Antioxidative defense of semen can be divided into those molecules belonging to sperm cells and those present in seminal plasma. The maintenance of redox balance is based on the concerted action of antioxidative enzymes, which mainly can act inside the cells, and non-enzymatic antioxidants present both in cells and body fluids, like seminal plasma. As sperm cells have small quantities of cytosol, in fact, they are not well protected from peroxidative damage and rely on seminal plasma antioxidants [3]. The source of ROS in seminal plasma is immature or abnormal sperm cells, where dysregulated mitochondria can produce them. In addition, leukocytes can produce certain quantities of ROS. These are necessary for appropriate capacitation, but when there are additional external sources of ROS, the imbalance between their production and ability to neutralize may lead to oxidative stress and serious problems in fertility, including inhibition of capacitation, DNA fragmentation, lipid peroxidation, decrease in membrane integrity, and decrease in motility due to the decrease in ATP. Damage to the sperm head leads to DNA fragmentation and infertility, while damage to the sperm tail cell membrane may lead to a decrease in motility and ability to fuse with an oocyte [1].

Out of the classic triad of antioxidative enzymes, SOD is responsible for the dismutation of two hydroperoxyl radicals into hydrogen peroxide and oxygen. Enzyme exists in a few isoforms, which differ in the structure of the active center and localization within the cell. Intracellular isoform located in cytosol and nucleus is a homodimer of 32 kDa and contains Cu, Zn in the active center, while the isoform located in mitochondria is a homotetramer of 96 kDa and contains Mn in the active center. Extracellular EC-SOD is a tetramer of 135 kDa and is located in plasma, lymph, synovial fluid, and seminal plasma [7].

SOD of sperm homogenates is sensitive to H_2_O_2_ inhibition, suggesting the CuZn isoform, but other isoforms are present as well. GSH-Px was confirmed in sperm cell homogenates [8] and most probably is more important for hydrogen peroxide decomposition than CAT. While some studies [9,10] found no detected activity of CAT in canine sperm cells, others reported its presence [11]. This discrepancy might be connected with different detection methods—zymography and spectrophotometry. Moreover, Angrimani et al. (2014) [11] examined the activity of SOD and GSH-Px in sperm cells collected from the caput, corpus, and cauda epididymides. The authors used spectrophotometric methods, while other authors mentioned in ejaculated sperm cell homogenates [12]. Dog semen has its own profile of enzymatic antioxidative defense [10,13].

Hydrogen peroxide—a product of the enzymatic activity of SOD—is further decomposed by both GSH-Px and/or CAT. GSH-Px can reduce hydrogen peroxide and other superoxides and exists in a few isoforms. Intracellular GSH-Px 1 is selenodependent, while extracellular GSH-Px3 is a selenoprotein. GSH-Px is supported by glutathione reductase (GR). CAT can simply degrade hydrogen peroxide and exists as a tetramer of 240 kDa.

Proteomic analysis of human sperm cells has been well described for many years, while such analysis in dogs is rare, mainly because interest in dog semen of dogs has only recently increased. The analysis of human sperm proteome by use of different proteomic techniques such as 2D electrophoresis, 2D-DIGE and further identification by MALDI or LC-MS/MS allowed for the description of even more than 1000 proteins out of which were also transcription factors and antioxidative proteins [14]. Protein profiles in human samples pointed out molecules involved in infertility, and further analysis of their abundance may find responsible genes and possible genetic disorders involved in mechanisms of infertility based on transcriptomic analysis [15]. Protein profiles identified in humans and other species may serve for a similar identification process in dogs. This is the reason why several omics can help solve problems of infertility, not only in humans but also in animals, including dogs.

Seminal plasma of dogs originates from the secretions of the prostate and epididymis. Proteins from this body fluid can bind sperm cells and facilitate different processes related to the transportation, capacitation, and fertilization. The comparison of sperm-rich and prostatic fractions by 1D electrophoresis showed the majority of similar proteins, including the most abundant lactotransferrin precursors and arginine esterase, while 17 proteins were sperm-rich specific [16]. This experiment confirms the lack of CAT in the seminal plasma of dogs. Lactotransferrin, as a molecule that transports iron, can play a role in the antioxidative defense of body fluids.

Kasimanickam et al. (2019) [17] compared proteins of seminal plasma (by 2D electrophoresis) of bulls characterized by high and low fertility and concluded that the differences in protein profile may interfere with fertility outcomes. Proteins which were characteristic for high fertility bulls are bovine seminal plasma (BSP1,3,5), spermadhesin 1, while for low fertility—clusterin (CLU), proteasome subunit alpha type 6 (PSMA6). These proteins are related to oxidative stress. BSP can modify cell membranes and may contribute to peroxidative damage, while CLU genes are known to be regulated by oxidative stress. The authors underlined posttranslational modifications as an important tool for the regulation of the biological activities of sperm and seminal plasma proteins [17].

The team of Gouletsou et al. (2022) evaluated the protein profile of sperm-rich and semen prostatic fractions in healthy dogs using sophisticated methods based on LC-MS/MS analysis on the LTQ Orbitrap Elite system and compared the obtained results with blood plasma proteins [18]. The authors found 59, 42, and 43 proteins in blood plasma, sperm-rich and semen prostatic fractions, respectively. There were a few common proteins in pairs of examined sources that may help to define specific markers of health or disease, as well as support transcriptomic analysis. Among identified proteins, there were no molecules directly related to oxidative stress or oxidative stress protection, which could be related to the used methods. Further elucidation is necessary with the use of other proteomic methods, as for sure more proteins are in blood plasma and should be in semen, even in comparison to human sperm [14]. Proteomic analysis can serve for quantitative analysis of enzymatic antioxidant proteins in sperm cells and seminal plasma.

Seminal plasma contains enzymatic antioxidants. Cassani et al. (2005) described for the first time SOD activity in canine seminal plasma by the spectrophotometric method and noticed a negative correlation between SOD activity and lipid peroxidation, and also the relations to sperm motility [8]. CAT and SOD activity in the seminal plasma of normal and asthenozoospermic dogs was detected by Kawakami et al. (2007) [19]. Neagu et al. 2011 confirmed the studies of Cassani et al. (2005) and added the studies on the presence of not only SOD but also GSH-Px in dog seminal plasma before and after thawing by use of spectrophotometric assays [8,20]. The authors concluded that the activity of these enzymes may influence sperm cell motility and velocity.

Non-enzymatic antioxidants, which can be divided into water-soluble, such as glutathione, uric acid, vit C, cysteine, bilirubin, carnosine, carnitine, Zn, selenium, as well as lipid-soluble, such as vitamin A, E, and derivatives of estrone and oestradiol, are present in seminal plasma [21]. Out of the list, vitamin C seems to be the most important, and physiological concentrations of vitamin C positively correlate with normal sperm cell morphology. Vitamin E cooperates with vitamin C in scavenging ROS and shows a relationship with the motility of sperm cells. In fact, all non-enzymatic antioxidants have their role in antioxidant defense, and their concentrations are diet dependent [22]. It clearly means that an appropriate diet is crucial for good fertility, which is often forgotten by owners of dogs [6]. It is especially important as these antioxidants are not renewable and should be supplemented constantly.

Enzymatic antioxidants are a renewable source of antioxidants and support non-enzymatic molecules in seminal plasma. They include SOD, CAT, GSH-Px, and GR. Their content and activity differ between species. Proteomic studies of seminal plasma allowed for the detection of haptoglobin and peroxiredoxin 4, which support protein enzymatic antioxidants. GSH-Px 3 can be considered a seminal plasma marker of male fertility potential. In accordance with Armstrong et al. (1998), albumin can exert antioxidative properties in seminal plasma [23]. Alpha-glucosidase is one of the possible markers of alterations in the human genital tract [24].

Total antioxidant capacity is a good laboratory tool for the estimation of antioxidative potential of seminal plasma, where all or almost all (depending on the method) non-enzymatic antioxidants can be measured [25]. For the appropriate interpretation of obtained results, not only the antioxidative but also the prooxidative activity and potential should be determined. The results should be compared within one patient over time rather than between species, as there are no reference values for TAC.

## 4. Impact of Oxidative Stress on Semen Quality and Fertility in Male Dogs

The impact of oxidative stress on semen quality is well described in humans. It is considered one of the main factors in infertility, regardless of the reasons for oxidative stress (internal, such as diseases; external, such as drugs, diet, and pollution) [26]. The authors claim that proteomic analysis of spermatozoa and seminal plasma could help determine not only the mechanisms of cell damage but also biomarkers of infertility. GSH-Px abundance was increased in samples with increased ROS content in comparison to healthy donors who also had lower ROS content. The authors suggested GSH-Px as a good biomarker of oxidative stress in human semen.

In the available literature, there are only a few studies on the relationship between oxidative stress and semen quality and fertility in male dogs (Table 1).

The cell membrane of dog sperm is rich in polyunsaturated fatty acids (PUFAs) [37], which makes it sensitive to oxidative damage by ROS. Damage to cell membrane lipids and fatty acids leads to changes in cell membrane permeability, which, together with protein peroxidation, also causes the lack of recognition of cells and the modification of antigenic properties of cells. Incubation of epididymal and ejaculated spermatozoa with various ROS showed that they were highly susceptible to hydrogen peroxide and hydroxyl radical but relatively more resistant to superoxide anion. Enzymes lose their activities due to changes in secondary structures. Oxidative phosphorylation in mitochondria is inhibited, collagen and hyaluronic acid are degraded and depolymerized, leading to changes in the cytoskeleton of cells. Finally, DNA can be fragmented and mutations may occur, causing malformation of fetuses, genetic disorders, and metabolic alterations. Finally, sperm motility is inhibited, and apoptosis is observed [1]. It seems that the composition of seminal plasma plays a key role in protecting sperm against oxidative stress. The sperm susceptibility to specific ROS was dependent on the presence of seminal plasma [28,29]. In fact, only small quantities of ROS are necessary for capacitation; when ROS excess occurs, capacitation is inhibited.

Treatment of dogs with dexamethasone to mimic stress significantly increased thiobarbituric acid-reactive substances (TBARS) in the seminal plasma, reduced ejaculate volume, and increased sperm morphological abnormalities [34]. In asthenozoospermic dogs, the activity of SOD and CAT in seminal plasma was significantly lower than in normospermic dogs [19]. Similarly, dogs with poor semen quality showed low SOD activity in seminal plasma [33]. It has been demonstrated that infertile dogs had poorer semen quality, lowered antioxidant status, and increased protein peroxidation in seminal plasma compared to fertile dogs [32]. Hypofertile dogs with no record of a live birth in the preceding year and poor semen quality showed higher serum ROS levels and oxidative stress index than fertile dogs [30]. However, some studies have found no differences between subfertile and fertile dogs in terms of sperm DNA peroxidation [27] and ROS production by sperm [31]. An individual effect in the susceptibility to the oxidative insult and a positive correlation between SOD activity in seminal plasma and post-thaw sperm motility were found [20].

Oxidative stress can also be induced by heat stress. In dogs under heat stress, an increase in serum oxidative stress parameters (TBARS and ROS), a decrease in antioxidant levels (SOD, CAT, GSH-Px, and TAC) were observed. This phenomenon was also observed at the spermatic level, with a marked increase in the production of ROS. Heat-induced oxidative stress had a detrimental effect on sperm concentration, motility, velocity, morphology, and viability [5].

A common cause of acquired infertility in male dogs is benign prostatic hypoplasia (BPH) [38,39]. This condition is associated with oxidative stress and lowered semen quality. Dogs with BPH induced by testosterone and oestradiol had reduced serum antioxidant enzyme activities [40]. Dogs with BPH showed lower serum total antioxidant activity (TAC) and an increasing trend in biomarkers of protein and lipid peroxidation in the serum compared to healthy dogs [41]. In dogs with BPH, a decrease in TAC and an increase in protein oxidation in prostatic fluid and spermatozoa were found. A higher proportion of sperm producing nitric oxide, a subclass of ROS, was reported in BPH dogs than in healthy dogs. The percentages of motile sperm, sperm with progressive motility, and normal sperm were lower in dogs with BPH than in non-affected dogs. The proportion of sperm in the motility subcategory STATIC was higher in dogs with BPH than in control dogs [35].

Semen quality in dogs declined with age [42]. Oxidative stress is believed to increase with age [43]. However, the decline in semen quality in aged dogs does not appear to be associated with increased susceptibility to oxidative stress [31,36,44].

Although the studies discussed above are relatively few in number, were conducted on a small number of animals, and used different methods, they indicate a link between oxidative stress and poor semen quality and reduced fertility in male dogs.

## 5. Effects of Antioxidant Supplementation of Dogs on Semen Quality and Fertility

The effect of various antioxidants on sperm quality and fertility in dogs has been investigated in several studies (Table 2).

Most studies have focused on vitamin E supplementation alone or in combination with other antioxidants. However, these studies yielded conflicting results. In one study, dogs were supplemented with vitamin E per os for 10 days to neutralize oxidation stress induced by dexamethasone treatment. Vitamin E oral supplementation reduced lipid peroxidation as indicated by the decrease in TBARS concentration in seminal plasma, increased sperm motility, vigor, and concentration, and decreased the percentage of major sperm defects [34]. Similarly, supplementation with vitamin E increased seminal plasma SOD activity and enhanced the sperm motility and the total sperm number in dogs with poor semen quality [45]. Daily oral supplementation of dogs with poor semen quality with vitamin E and essential fatty acids (omega 3, 6, and 9) for 60 days significantly increased ejaculate volume and cell vigor and decreased the number of morphologically abnormal sperm [46]. However, another study found no effect of oral supplementation with vitamins E and C for 60 days on sperm DNA peroxidation, measured by the concentration of 8-hydroxy-2′-deoxyguanosine, in dogs with reduced semen quality and fertility [27]. Supplementation of the diet of normospermic dogs with fish oil rich in omega 3-n fatty acids for 120 days increased percentages of motile sperm and morphologically normal sperm, sperm count, and sperm viability. Supplementation of dogs with fish oil alone or together with vitamin E decreased lipid peroxidation in sperm samples peroxidized by adding ascorbate–Fe^2+^ [47]. Supplementation with Se and vitamin E for a period of 60 days enhanced the antioxidant status of sperm and improved the quality of semen in dogs with lowered fertility. After supplementation, GSH-Px activity and TAC in sperm, concentration of spermatozoa, the majority of motility indicators, and percentage of normal morphology and live spermatozoa increased significantly [25]. In four infertile dogs, such supplementation restored fertility [32]. On the contrary, Kirchhoff et al. [42] failed to identify a clear trend about how a 3 month vitamin E and/or Se supplementation affects semen quality in normospermic dogs, although an effect of treatment could be found for the percentage of sperm head defects. The study of Alonge et al. [44] investigated the effect of daily supplementation with a complex of vitamin E, zinc, selenium, folic acid, and n-3 PUFAs on semen quality in normospermic dogs [49]. The supplementation significantly improved total sperm count, progressive motility, functional membrane integrity, and sperm viability.

Supplementation with other antioxidants in dogs was also investigated. Oral supplementation of dogs with poor semen quality with coenzyme Q10 (ubiquinol) once daily for 12 weeks improved sperm motility, reduced morphologically abnormal sperm, and increased seminal plasma SOD activity [33]. Dietary supplementation of subfertile dogs for 62 days with extract from Maca (*Lepidium meyenii*), an Andean crop with antioxidant activity, resulted in a significant increase in ejaculate volume, total sperm count, total and progressive motility, and sperm morphology [50], as well as better preservation of semen quality during cold storage [51]. Recently, it was reported that supplementation of dogs exposed to natural heat stress with polyphenolic extract from Loblolly pine (Pinus taeda) lignin for 90 days enhanced antioxidant defense and mitigated oxidative damage of sperm. The supplemented dogs showed higher progressive sperm motility and a greater percentage of rapid-movement sperm [52].

The studies’ analyses were conducted on a small number of animals and showed significant methodological differences in terms of the type of antioxidant, dosage, duration of use, and assessment of oxidative status. Only a few studies evaluated the effect of antioxidant supplementation on fertility. Nevertheless, these studies indicate that antioxidant supplementation improves the oxidative status and quality of semen and may have a positive effect on the fertility of male dogs with reproductive problems.

Furthermore, it has been suggested that antioxidant supplementation may help restore sperm quality in men with reproductive system pathologies such as BPH and inflammation (prostatitis, epididymitis, orchitis) [53,54]. These diseases in dogs are associated with oxidative stress and impaired spermatogenesis [41,55]; therefore, the use of antioxidants seems justified.

## 6. Effects of Antioxidant Supplementation to Semen Extenders on Antioxidant Status, Semen Quality, and Fertility in Male Dogs

Handling and preservation of canine semen may generate sperm damage and reduced fertilization capacity as a consequence of reactive oxygen species formation [56]. Cryopreservation of semen avoids the transfer of some diseases and allows breeding of dogs living far away from each other or overcoming mating inability, but carries the danger of alterations in semen quality related to the freezing and thawing process. Semen can be chilled up to 4 °C or frozen. Canine sperm are particularly sensitive to cryopreservation, which leads to increased formation of ROS and lipid peroxidation of plasma membranes that contain large amounts of PUFAs [37]. Damage is related to reactive oxygen species, which are generated in excess during thawing and cause osmotic changes. Moreover, changes in the lipid phase of cell membranes connected to alterations in cholesterol and fatty acid content occur. Changes in the structure of the cell membrane may influence capacitation-like changes due to an increase in intracellular calcium and an increase in protein phosphorylation [57]. Furthermore, the removal of seminal plasma prior to cryopreservation radically reduces the availability of antioxidants [58]. A number of studies have been performed on the addition of various antioxidants to the canine semen extenders in order to mitigate the impact of oxidative stress on the sperm (Table 3). However, some findings have been inconsistent between the studies.

Supplementation of enzymatic (CAT) and non-enzymatic antioxidants (vitamin C, vitamin E, NAC) to semen extender has been shown to improve dog semen quality after cryopreservation [59], and short and long-term cold storage [60]. Addition of vitamin E to canine semen extender with DEHA improved sperm motility characteristics after cryopreservation, whereas addition of 300 U/mL catalase plus DEHA had a deleterious effect on mitochondrial activity [61]. Supplementation of semen extender with SOD plus GSH-Px enhanced the viability and DNA integrity of cold-stored and frozen–thawed canine sperm [62]. Addition of SOD, CAT, and GSH-Px combination to semen extender increased total and progressive motilities and DNA integrity of sperm of fertile and hypofertile dogs during 10 days of storage at 4 °C [30]. The presence of melatonin in semen extender did not affect DNA fragmentation, motility, and acrosome integrity of epididymal sperm [63]. However, supplementation of melatonin to the freezing and cooling media improved motility, viability, acrosome, and DNA integrity of ejaculated canine sperm [64,65].

The results of studies on reduced glutathione (GSH) supplementation to semen extenders are contradictory. In one study [66], supplementation of semen extender was effective in improving semen quality. Similarly, the addition of GSH to semen extender resulted in acrosome protection [67]. However, another study found no effect of supplementation with different concentrations of GSH on the quality of chilled and thawed semen. The higher concentrations of GSH had deleterious effects on mitochondrial activity post-thaw [68]. No overall positive effect was observed for GSH addition on sperm motility in chilled dog semen samples during 4- and 10-day of storage [69].

Supplementation of canine semen with curcumin, a yellow pigment from curcuma with antioxidant properties, to semen extender ameliorated sperm total count, motility, progressive motility, abnormality, DNA integrity, TAC, and mRNA expression of NADPH oxidase 5 (NOX5) gene [70]. The addition of 1 mg/mL of myo-inositol, a natural compound with antioxidant properties, to semen extender had beneficial effects on the post-thaw dog sperm motility, viability, plasma membrane integrity, and chromatin integrity. Improvement in post-thaw semen quality was confirmed by the expression of genes related to apoptosis, nuclear integrity, and reactive oxygen species generation [71]. Supplementation of semen extender with iodixanol, a radiographic agent with the potential to reduce oxidative stress in biological systems, decreased ROS production and prevented detrimental effects during the cryopreservation of dog semen, as evidenced by increased motility and reduced cryocapacitation in frozen and thawed spermatozoa [72]. Spermine, a natural polyamine acting as an antioxidant, added to semen, reduced ROS production, prevented apoptosis and adverse effects of cryocapacitation during canine sperm cryopreservation, and improved post-thaw kinematic parameters and membrane integrity of sperm [73]. The supplementation of astaxanthin, a keto-carotenoid with higher antioxidant activity, to semen extender resulted in improved freeze–thaw sperm quality of the dog. In comparison with the control group, sperm samples supplemented with astaxanthin showed significantly higher sperm counts with intact membranes, intact acrosomes, active mitochondria, and normal chromatin. Furthermore, astaxanthin-supplemented samples showed significantly lower expression levels of pro-apoptotic (BAX), oxidative-induced DNA damage repair (OGG1), oxidative stress-related (ROMO1) genes, and higher expression levels of anti-apoptotic (BCL2), and sperm acrosome-associated (SPACA3) genes compared to the control [71]. Dog sperm cryopreserved with resveratrol, a non-flavonoid powerful antioxidant, showed significant improvement in post-thaw sperm motility and viability, higher numbers of sperm with an intact plasma membrane, active mitochondria, and structural integrity of acrosomes and chromatin compared with that of the control group [75]. Addition of quercetin, a plant flavonoid with strong antioxidant effects, to cryopreservation solutions was reported to improve post-thaw total motility of canine sperm. Three bitches were transcervically inseminated with cryopreserved sperm treated with quercetin. All the bitches became pregnant and delivered a total of 18 puppies [76]. Adding kinetin, a cytokinin which may act as an antioxidant, to semen extender improved motion characteristics and viability of post-thaw sperm samples. Kinetin-supplemented samples exhibited higher sperm counts with the intact plasma membrane, normal acrosomes, mitochondria, and chromatin than controls [77]. Supplementation by metformin, a molecule that limits oxidative stress, to the dog semen diluent reduced ROS production and improved sperm motility after thawing compared to the control group. The improvement in semen quality was confirmed using various molecular markers (phospho-AMPK protein, Hsp70, and phospho-ERK) [78]. The addition of lycopene, a potent carotenoid antioxidant, to semen extender improved canine semen parameters and TAC levels and decreased malondialdehyde (MDA) levels in the chilling process [79]. Supplementation with 3,4-dihydroxyphenyl glycol, a phenolic olive-derived antioxidant, to canine semen extender improved oxidative markers (TAC, GSH, antioxidant enzymes SOD, CAT, GSH-Px), the motility characteristics and plasma membrane integrity, and reduced DNA damage in the cryopreservation process [80]. Addition of ergothioneine, an unusual thio-histidine betaine amino acid and potent antioxidant that is synthesized by microbes, to canine semen extender increased post-thaw motility characteristics and acrosome integrity, and reduced morphological alterations. Isoespintanol, an extract from *Oxandra* cf. *xylopioides* (*Annonaceae*) leaves, was less efficient, as it did not affect sperm motility [81]. Supplementation with procyanidin, a polyphenolic compound with antioxidant activity, to canine semen extender increased TAC levels and expression of SOD, CAT, and GSH-Px genes in sperm. Plasma membrane integrity, acrosome integrity, and mitochondrial membrane potential of sperm stored at 4 °C were higher than the control group [82]. Addition of N-acetyl cysteine, a precursor of glutathione, to semen extender did not affect ROS production, but increased the motility of chilled canine sperm [83]. In another study, increased post-thaw motility, viability, and membrane integrity of canine sperm after supplementation with N-acetyl cysteine were found [84].

In conclusion, many studies have been conducted on the supplementation of various endogenous and exogenous antioxidants to canine semen extenders, but the results are inconclusive. This may be due to differences in the semen extenders, antioxidant dosages, and oxidative stress markers. Most studies point to the potential role of antioxidant supplementation in extenders in protecting canine sperm from oxidative damage during processing. However, the final endpoint for assessing the fertilizing capacity of canine semen remains the pregnancy rate. Only a few studies have assessed the fertilization capacity of supplemented sperm after AI. Therefore, further research is needed to assess the impact of adding antioxidants to semen extenders on canine fertility.

## 7. Conclusions

Dog semen has an antioxidant defense system. However, an imbalance between the production and neutralization of ROS can lead to oxidative stress. This plays an important role in the pathogenesis of fertility disorders in male dogs. Oral antioxidant supplementation improves the oxidative status and quality of semen and may have a positive effect on the fertility of male dogs with reproductive problems. Dog semen is sensitive to oxidative damage during handling and storage. Adding antioxidants to dog semen diluents may preserve semen fertility. Further studies with larger sample sizes, taking into account fertility outcomes, are needed to investigate the usefulness of antioxidants in the supportive treatment of fertility disorders and sperm fertility preservation.

## Figures and Tables

**Table 1:** Oxidative stress and semen quality variables and fertility in dogs.

Oxidative Stress Variables	Semen Quality Variables
Lower SOD and CAT in seminal plasma in asthenozoospermic dogs than in normospermic dogs.	Asthenozoospermia (percentage of actively motile sperm less than 50%) [19].
SOD and GPx activity in seminal plasma, lipid peroxidation of sperm.	Positive correlation of SOD activity with sperm motility and negative correlation of GPx activity with sperm viability post-thaw. An individual effect on sperm lipid peroxidation [20].
No differences between subfertile and fertile dogs in sperm DNA peroxidation measured by 8-hydroxy-2′-deoxyguanosine concentration.	Low sperm count and/or more than 30% of total sperm pathology in subfertile dogs [27].
Ejaculated and epididymal semen samples (with or without seminal plasma) were incubated with ROS generation systems (superoxide anion [O_2_^−^], hydrogen peroxide [H_2_O_2_], and hydroxyl radical [OH^−^] and malondialdehyde [MDA]).	In ejaculated semen, H_2_O_2_ reduced mitochondrial membrane potential, integrity of plasma, and acrosome membrane integrity and sperm kinetics in the absence of seminal plasma. OH^−^ reduced mitochondrial activity and increased DNA fragmentation independent of the absence or presence of seminal plasma. O_2_^−^ decreased the mitochondrial activity in the absence of seminal plasma [28]. Corpus and cauda epididymal sperm were highly susceptible to the deleterious effects of H_2_O_2_, OH^−^, and MDA. Epididymal canine sperm are relatively resistant to O_2_^−^ damage [29].
Higher serum ROS level and oxidative stress index in hypofertile than in fertile dogs.	Low volume and sperm motility, increased morphological alteration in hypofertile dogs [30].
No differences in sperm ROS production between fertile and subfertile dog.	Lower viability and percentage of normal sperm in frozen-thawed semen in subfertile dogs than in fertile dog [31].
Lower TAC, higher contents of protein peroxidation markers in seminal plasma in infertile than in fertile dogs.	Low sperm concentration, total sperm count, motility parameters, and the percentage of sperm with normal morphology in infertile dogs [32].
Low SOD activity in seminal plasma in dogs with poor semen quality.	Low total number of sperm and percentage of progressively motile sperm in semen [33].
Increased TBARS level in seminal plasma after treatment with dexamethasone.	Reduced ejaculate volume and increased sperm morphological abnormalities [34].
Increased serum TBARS and ROS, decreased SOD, CA, GPx, and TAC in dogs under heat stress.	Decreased sperm concentration, motility, velocity, morphology, and viability [5].
A higher proportion of sperm producing NO in BPH dogs than in healthy dogs.	Decreased motility parameters and the percentage of normal sperm [35].
No differences in sperm lipid peroxidation and activities of SOD, CAT, and GPx in seminal plasma and sperm.	Lower total motility, sperm concentration, total sperm count, membrane integrity, and zona binding capacity in senile dogs compared to mature dogs [36].

**Table 2:** Effects of antioxidants supplementation of dogs on semen quality, antioxidant status, and fertility.

Antioxidant	Supplementation	Effects
Vitamin E	500 mg per os for 10 days in 5 dogs with oxidation stress induced by dexamethasone treatment 50 mg per os for 4 weeks in 4 dogs with poor semen quality	Antioxidant status: reduced lipid peroxidation (decreased TBARS in seminal plasma). Semen quality: increased sperm motility, vigor, and concentration, and decreased percentage of major sperm defects. Fertility: n.a. [34]. Antioxidant status: increased seminal plasma SOD activity. Semen quality: enhanced sperm motility and total sperm number. Fertility: n.a. [45].
Essential fatty acids (omega 3, 6, and 9) and vitamin E	Food supplemented with linoleic acid (omega 3)—7.2 mg per body weight (BW), linolenic acid (omega 6)—25 mg ⁄kg, oleic acid (omega 9)—10.1 mg⁄kg, and vitamin E—1 UI/kg for 60 days in 8 healthy dogs.	Antioxidant status: n.a. Semen quality: increased ejaculate volume and cell vigor, and decreased the number of morphologically abnormal sperm. Fertility: n.a. [46].
Vitamin C and E	500 mg vitamin C and 500 mg vitamin E per os for 60 days in 5 fertile and 6 subfertile dogs	Antioxidant status: no effect on sperm DNA peroxidation. Semen quality: n.a. Fertility: n.a. [27].
Fish oil or fish oil and vitamin E	54 mg fish oil/kg metabolic body weight or 54 mg fish oil/kg metabolic BW plus 400 mg vitamin E for 60 days	Antioxidant status: decreased lipid peroxidation in sperm samples peroxidized by adding ascorbate–Fe^2+^. Semen quality: increased percentage of motile sperm, total sperm count, total sperm viability, and total morphologically normal sperm.Fertility: n.a. [47].
Se and vitamin E	Se (0,6 mg/kg organic Se yeast) and vitamin E (5 mg/kg) for a period of 60 days in 10 dogs with lowered fertility. Se (0.6 mg/kg organic Se yeast) and vitamin E (5 mg/kg) for a period of 60 days in 4 infertile dogs.	Antioxidant status: increased serum concentration of Se and vitamin E, and GSH-Px-activity and TAC in the spermatozoa. Semen quality: increased concentration of sperm, motility indicators, and percentages of normal morphology and live sperm. Fertility: n.a. [25]. Antioxidant status: increased serum concentration of Se and vitamin E. Semen quality: increased motility parameters, percentages of live and normal morphology sperm. Fertility: 4 bitches became pregnant after AI [32].
Se, vitamin E or Se + vitamin E	0.1 mg Se, 100 mg vitamin E, or 0.1 mg Se plus 100 mg vitamin E in 3 normospermic dogs each.	Antioxidant status: high variation in GSH-Px in blood and seminal plasma. Semen quality: decreased percentage of sperm head defects. Fertility: n.a. [48].
Fish oil	Fish oil 54 mg/kg metabolic BW for 120 days in 5 healthy dogs.	Antioxidant status: n.a. Semen quality: increased percentage of motile sperm, total sperm count, total sperm viability, and total morphologically normal sperm. Fertility: n.a. [47].
Complex of vitamin E, zinc, selenium, folic acid, and n-3 polyunsaturated fatty acids	Vitamin E (5 mg/kg BW), zinc (3 mg/kg BW), Se (0.007 mg/kg BW), folic acid (0.625 mg/kg BW). Refined fish oil (25% DHA and 10% eicosapentaenoic acid [EPA]) in 14 normospermic dogs for 90 days.	Antioxidant status: n.a. Semen quality: total sperm count, progressive motility, functional membrane integrity, and sperm viability. Fertility: n.a. [49].
Ubiquinol	100 mg of ubiquinol orally once daily for 12 weeks in three dogs with poor semen quality.	Antioxidant status: increased seminal plasma SOD activity Semen quality: improved sperm motility and reduced morphologically abnormal sperm.Fertility: n.a. [33].
Extract from Maca (*Lepidium meyenii*)	75 mg/kg for 62 days in 12 subfertile and 12 fertile dogs for 62 days. 75 mg/kg for 62 days in 10 subfertile and 10 fertile dogs for 62 days.	Antioxidant status: n.a. Semen quality: increased ejaculate volume, total sperm count, total and progressive motility, and sperm morphology in subfertile dogs. Fertility: n.a. [50]. Antioxidant status: n.a. Semen quality: increased ejaculate volume, concentration, motility, morphology, and sperm membrane integrity in subfertile and fertile dogs. Better preservation of semen quality during storage at 5 °C [51].
Extract from Loblolly pine (*Pinus taeda*) lignin	50 mg/kg/day orally in 20 healthy dogs for 120 days.	Antioxidant status: Semen quality: higher progressive sperm motility and a greater percentage of rapid-movement sperm. Fertility: n.a. [52].

n.a.—not analyzed.

**Table 3:** Effects of antioxidants supplementation to semen extenders on antioxidant status, semen quality, and fertility in male dogs.

Antioxidant	Supplementation	Effects
vitamin C NAC taurine CAT vitamin E vitamin B16	1.5 mM 1.5 mM 0.6 mM 300 U/mL 0.3 mM 0.3 mM	Antioxidant status: no effect on ROS production. Semen quality: most pronounced effect of CAT on semen quality—increased post-thaw motility, percentages of rapid motile and viable sperm. B16 addition had adverse effects on semen quality. Fertility: n.a. [59].
vitamin C NAC taurine CAT vitamin E vitamin B16	0.5 mM 0.5 mM 0.2 mM 100 U/mL 0.1 mM 0.1 mM	Antioxidant status: no effect on ROS production. Semen quality: the most pronounced effect of vitamin E and B16 on semen quality—increased motility, percentages of rapid motile and viable sperm after short and long-term cold storage. Fertility: n.a. [60].
Vitamin E plus DHA CAT plus DHA Vitamin E plus CAT plus DHA	0.6 mM vitamin E plus 5 µM DHA 300 U/mL CAT plus 5 µM DHA 0.6 mM vitamin E plus 300 U/mL CAT plus 5 µM DHA	Antioxidant status: lower levels of oxidative stress in treatment groups. Semen quality: improved sperm motility characteristics after addition of vitamin E plus DHA. The CAT plus DHA group was harmful to sperm mitochondria. Fertility: n.a. [61].
SOD plus GPx	100 IU SOD plus 5 IU GPx	Antioxidant status: no effect on SOD and GPx activities during cold storage, decreased SOD activity in frozen-thawed samples after cold storage, and no differences in ROS levels. Semen quality: increased percentages of sperm viability and DNA integrity after cold storage and freezing-thawing. Fertility: n.a. [62].
Combination of SOD, CAT, and GPx	5 IU/mL of GPx, 15 IU/mL of CAT, and 15 IU/mL of SOD.	Antioxidant status: n.a. Semen quality: increased sperm motility and DNA integrity in semen of fertile and hypofertile dogs after 5 and 10 days of cooled storage. Fertility: n.a. [30].
	1 mM	Antioxidant status: n.a. Semen quality: DNA fragmentation, motility, and acrosome integrity of epididymal sperm. Fertility: n.a. [63].
Melatonin	0.0005, 0.002, and 0.0035 mol/L 1 and 2 mM	Antioxidant status: n.a. Semen quality: 0.002 and 0.0035 mmol/L decreased percentage of sperm having hyper-fluid membranes, increased percentages of intact acrosome, capacitated acrosome-intact, and acrosome-reacted of post-thaw sperm cooled to −5 °C before freezing. Fertility. n.a. [64]. Antioxidant status: Semen quality: increased motility, viability, acrosome, and DNA integrity during cooling storage. Increased post-thaw sperm DNA integrity. Fertility: n.a. [65].
	2.5, 5, 7.5, and 10 mM	Antioxidant status: decreased lipid peroxidation (malondialdehyde concentration) after thawing in the 5 and 10 mM GSH groups. Semen quality: 5 and 10 mM GSH increased post-thaw motility, viability, and acrosome integrity. Fertility: AI with semen of the 5 mM GSH group in 4 bitches resulted in five puppies from two bitches [66].
	10 and 20 mM	Antioxidant status: increased TBARS after thawing in the GSH-20 group. Semen quality: 20 mM GSH promoted post-thaw sperm damage, especially to mitochondrial activity. A total of 10 mM GSH resulted in acrosome protection Fertility: Three bitches became pregnant after AI with semen cryopreserved in extender with 10 mM GSH [67].
GSH	5, 7.5, 10 mM	Antioxidant status: no effect on TBARS. Semen quality: A total of 5 mM of GSH improved mitochondrial activity in chilled and thawed samples; higher concentrations (7.5 and 10 mM) decreased mitochondrial activity in chilled and thawed samples. Thawed samples of 10 mM of GSH had high DNA fragmentation rates. Fertility: n.a. [68].
	5 and 10 mM	Antioxidant status: n.a. Semen quality: no general positive effect of GSH addition on values for chilled semen variables during storage for as long as 10 days. Fertility: n.a. [69].
Curcumin	2.5 mM	Antioxidant status: increased TAC and mRNA expression of the NADPH oxidase 5 gene. Semen quality: increased sperm total count, motility, progressive motility, and DNA integrity. Lower percentage of abnormal sperm. Fertility: n.a. [70].
Myo-inositol	1 mg/mL	Antioxidant status: n.a. Semen quality: increased post-thaw dog sperm motility, viability, plasma membrane integrity, and chromatin integrity. Fertility: n.a. [71].
Iodixanol	1.5%	Antioxidant status: reduced ROS production. Semen quality: increased post-thaw motility and reduced cryocapacitation. Fertility: n.a. [72].
Spermine	5.0 mM	Antioxidant status: reduced ROS production. Semen quality: reduced apoptosis and cryocapacitation, increased post-thaw kinematic parameters, and membrane integrity of sperm. Fertility: n.a. [73].
Astaxanthin	1 µM	Antioxidant status: n.a. Semen quality: higher sperm counts with intact membranes, intact acrosome, active mitochondria, and normal chromatin. Fertility: n.a. [74].
Resveratrol	200 µM	Antioxidant status: n.a. Semen quality: increased post-thaw sperm motility and viability, higher numbers of sperm with an intact plasma membrane, active mitochondria, and structural integrity of acrosomes and chromatin. Fertility: n.a. [75].
Quercetin	5 mg/mL	Antioxidant status: n.a. Semen quality: increased motility of cryopreserved canine sperm after thawing. Fertility: three bitches became pregnant after AI [76].
Kinetin	5.0 mM	Antioxidant status: n.a. Semen quality: increased motility, viability, sperm counts with the intact plasma membrane, normal acrosomes, mitochondria, and chromatin. Fertility: n.a. [77].
Metformin	50 µM	Antioxidant status: reduced ROS production. Semen quality: increased post-thaw sperm motility. Fertility: n.a. [78].
Lycopene	250, 500, and 750 µg/mL	Antioxidant status: 500 µg/mL lycopene increased TAC and decreased MDA levels in chilled canine semen. Semen quality: 500 µg/mL lycopene increased motility characteristics, viability, and hypo-osmotic swelling test (HOST) percentages of canine semen preserved at 5 °C for 72 h. Fertility: n.a. [79].
3,4-dihydroxyphenyl glycol	50 µg/mL	Antioxidant status: increased TAC, GPx, and GSH. Semen quality: increased post-thaw motility characteristics, plasma membrane integrity, and reduced DNA damage. Fertility: n.a. [80].
Ergothioneine	50, 100, and 150 mM	Antioxidant status: 100 mM reduced ROS production after thawing. Semen quality: 100 mM increased post-thaw motility characteristics and acrosome integrity. Fertility: n.a. [81].
Isoespintanol	20, 40, 60 mM	Antioxidant status: 40 and 60 mM reduced ROS production after thawing. Semen quality: 60 mM reduced morphological alterations and increased acrosome integrity of thawed canine spermatozoa. Fertility: n.a. [81].
Procyanidin	30 μg/mL	Antioxidant status: increased TAC levels and expression of SOD, CAT, and GPx genes in sperm. Semen quality: Increased plasma membrane integrity, acrosome integrity, and mitochondrial membrane potential of sperm stored at 4 °C. Fertility: n.a. [82].
	0.5 mM	Antioxidant status: no effect on ROS production. Semen quality: increased motility of chilled canine sperm. Fertility: n.a. [83].
NAC	0.35 mM	Antioxidant status: n.a. Semen quality: increased post-thaw motility, viability, and membrane integrity of canine sperm. Fertility: n.a. [84].

NAC—N-acetyl cysteine, DEHA—docosahexaenoic acid, n.a.—not analyzed.

## Data Availability

The original contributions presented in this study are included in the article. Further inquiries can be directed to the corresponding author.
